# Effects of gastrointestinal symptoms on the efficacy of washed microbiota transplantation in patients with autism

**DOI:** 10.3389/fped.2025.1528167

**Published:** 2025-02-13

**Authors:** Dong-Xia Hu, Cai-Mei Lu, Xin-Yu Si, Qing-Ting Wu, Li-Hao Wu, Hao-Jie Zhong, Xing-Xiang He

**Affiliations:** ^1^ Department of Gastroenterology, Research Center for Engineering Techniques of Microbiota-Targeted Therapies of Guangdong Province, The First Affiliated Hospital of Guangdong Pharmaceutical University, Guangzhou, China; ^2^ Department of Hepatobiliary and Pancreatic Surgery, The First Affiliated Hospital of Shenzhen University, Shenzhen Second People’s Hospital, Shenzhen, China; ^3^ Guangdong Provincial Key Laboratory for Research and Evaluation of Pharmaceutical Preparations, Guangdong Provincial Engineering Center of Topical Precise Drug Delivery System, Guangdong Pharmaceutical University, Guangzhou, China

**Keywords:** autism, washed microbiota transplantation, clinical efficacy, gastrointestinal symptoms, treatment course

## Abstract

**Objective:**

Washed microbiota transplantation (WMT) has emerged as a promising therapeutic strategy for autism spectrum disorder (ASD), though the factors that influence its efficacy remain poorly understood. This study explores the impact of gastrointestinal (GI) symptoms on the effectiveness of WMT in ASD.

**Methods:**

Clinical data encompassing ASD symptoms, GI disturbances, and sleep disorders were collected from patients with ASD undergoing WMT. The therapeutic impact of WMT and the contributing factors to its efficacy were assessed.

**Results:**

WMT significantly reduced scores on the Aberrant Behavior Checklist (ABC), Childhood Autism Rating Scale (CARS), and Sleep Disturbance Scale for Children (SDSC), alongside a significant reduction in the incidence of constipation, abnormal stool forms, and diarrhea (all *p* < 0.05). After six courses of WMT, substantial reductions were observed in ABC, CARS, and SDSC scores, with increased treatment courses correlating with greater improvement (*p* < 0.05). Multiple linear regression analysis revealed that WMT efficacy was enhanced in patients with pre-existing GI symptoms (diarrhea: *β* = 0.119, *p* < 0.001; abnormal stool form: *β* = 0.201, *p* < 0.001) and those receiving a higher number of treatment courses (*β* = 0.116, *p* < 0.001). Additionally, the analysis indicated that treatment outcomes were more favorable in patients who had not undergone adjunct interventions (*β* = −0.041, *p* = 0.002), had a longer disease duration (*β* = 0.168, *p* = 0.007), and exhibited more severe disease symptoms (*β* = 0.125, *p* < 0.001).

**Conclusion:**

WMT significantly alleviates both ASD and GI symptoms, along with sleep disturbances, in affected individuals. Six treatment courses resulted in notable improvement, with increased course numbers further improving therapeutic outcomes. Furthermore, pre-treatment GI symptoms, such as diarrhea and abnormal stool forms, may influence the effectiveness of WMT. Notably, patients who did not receive additional interventions, had a prolonged disease duration, and presented with more severe symptoms experienced markedly improved treatment responses.

## Introduction

Autism spectrum disorder (ASD) is a multifaceted neurodevelopmental condition that impacts the central nervous system, resulting in impairments in communication, behavior, and social interactions (1). The global prevalence of ASD was approximately 1% in 2022 (2), with a continually rising incidence, and a male-to-female ratio of 4–5:1 (3). Despite this prevalence, no definitive cure for ASD currently exists.

The microbiota-gut-brain axis (MGBA) represents the complex bidirectional communication between the brain and the gastrointestinal (GI) tract (4). The stability of the gut microbiota plays a pivotal role in this axis, as dysbiosis can negatively affect not only the GI system but also psychiatric health (5, 6). Patients with ASD often present with disturbances in gut microbiota composition (7), and research suggests that fecal microbiota transplantation (FMT) may alleviate some of the symptoms of ASD (8, 9, 10). Washed microbiota transplantation (WMT), an advanced form of FMT (11), involves microfiltration of microbiota preparations, utilizing an automated purification system to remove fecal particles, parasite eggs, and fungi. This process also eliminates metabolites with pro-inflammatory effects from the fecal microbiota supernatant, resulting in fewer FMT-related adverse events compared to traditional manual FMT preparation (12). Furthermore, WMT has been shown to improve symptoms of ASD (13, 14). However, its therapeutic efficacy remains variable among patients with ASD.

GI symptoms are prevalent comorbidities in ASD, including diarrhea, constipation, and abnormal stool forms (15, 16), with the incidence of these symptoms being six times higher in children with ASD compared to their typically developing peers (17). These GI disturbances can exacerbate behavioral issues, such as rigidity, hyperactivity, and social withdrawal (18, 19). Moreover, the severity of symptoms and disease duration have been shown to influence the effectiveness of FMT in conditions like ulcerative colitis (UC) and inflammatory bowel disease (IBD) (20, 21). Given these considerations, the present study aims to investigate the factors that influence the effectiveness of WMT in ASD treatment, with a particular focus on GI symptoms.

## Materials and methods

### Patient selection and ethics statement

Patients from the First Affiliated Hospital of Guangdong Pharmaceutical University who met the DSM-5 criteria for a diagnosis of ASD and received at least two consecutive WMT courses between June 2019 and June 2024 were recruited for the study. Inclusion criteria were as follows: (1) age between 2 and 18 years; (2) confirmed ASD diagnosis; (3) signed informed consent from the patients’ parents or guardians; and (4) willingness of the patients' parents or guardians to complete questionnaires related to ASD and GI symptoms. Exclusion criteria included: (1) severe heart, lung, liver, or kidney disease; (2) a history of brain injury, cerebral palsy, head tumors, or major brain malformations; and (3) participation in other clinical trials within the last month. The study was approved by the institutional review board of our hospital (#2020-14). Informed consent was obtained from the parents or guardians of all included patients before enrollment.

### Clinical protocol of WMT

Healthy donors underwent thorough screening for WMT, as previously outlined (13). This screening process included a questionnaire and a physical examination by a clinician. Stool and blood samples were tested to rule out any potential infectious or communicable diseases (22). Informed consent was also obtained from the parents or guardians of the healthy donors before initiating the WMT procedures. WMT was routinely administered to hospitalized patients at our institution using fresh microbiota, as described in prior research (23). In brief, a homogeneous fecal suspension was prepared with a ratio of 100 g of feces to 500 ml of normal saline. The fecal suspension was then filtered using a microfiber filtration instrument (GenFMTer; FMM Medical, Nanjing, China) to remove food particles, inflammatory substances, and fungi. This process enhances the safety of the microbiota suspensions. Each WMT treatment involved the administration of a fecal suspension containing approximately 5.0 × 10^13^ bacteria, delivered daily (60–90 ml) for 6 consecutive days via a transendoscopic enteral tube. According to the Nanjing Consensus on the Methodology of Washed Microbiota Transplantation (24), only fresh, healthy microbiota were used, with preparation and transplantation completed within 1 h. Patients were instructed to remain in the left lateral position for 2 h post-injection and to consume light meals for at least 2 days after WMT. Follow-up WMT treatments were scheduled monthly.

### Data collection

Patients were assessed at baseline, prior to the first course of WMT treatment, and before each subsequent course. Clinical characteristics were gathered from the patients' electronic medical records. The following questionnaires were employed to assess ASD- and GI-related symptoms. The Aberrant Behavior Checklist (ABC) (25) is a validated and reliable instrument widely used to assess ASD symptoms (26). It consists of 57 items across five key domains: stereotypy, irritability, lethargy, inappropriate speech, and hyperactivity. The Childhood Autism Rating Scale (CARS) (27) is a 15-item tool used to diagnose ASD and assess the overall severity of symptoms. Patients with scores between 30 and 36 are considered to have mild to moderate symptoms, while those with scores greater than 36 are categorized as having severe symptoms. The CARS is completed by pediatricians or other qualified professionals (28). The Sleep Disturbance Scale for Children (SDSC) is a 34-item questionnaire completed by parents, with scores ranging from 34 to 170. Scores above 39 suggest the presence of a sleep disorder (29). The Bristol Stool Form Scale (BSFS) is used to evaluate stool consistency (30), where scores of 1 or 2 indicate hard stool, 6 or 7 represent soft stool, and scores of 3–5 reflect normal stool consistency. The Rome III criteria were applied to define constipation (31), while diarrhea was characterized by the occurrence of three or more loose or watery stools (scores of 5–7 on the BSFS) per day.

### Statistical analysis

All statistical analyses were performed using SPSS 23.0 (IBM Corp., Armonk, NY, United States) and Prism 8 software (GraphPad Prism Inc., San Diego, CA, United States). Continuous data were expressed as means (standard deviation) or medians (interquartile range), depending on the distribution, while categorical data were presented as *n* (%). Categorical variables were analyzed using Chi-square and Fisher's exact tests. The normality of continuous variables was tested using the Shapiro–Wilk test. For comparing differences in continuous variables between independent groups, the unpaired Student's *t*-test (for normal distribution) or Mann–Whitney *U*-test (for non-normal distribution) was applied. Paired data were analyzed using the paired Student's *t*-test (for normal distribution) or Wilcoxon signed-rank test (for non-normal distribution). Multivariate linear regression analysis was conducted to identify potential influencing factors. A *p*-value of less than 0.05 was considered statistically significant.

## Results

### Characteristics of the patients With ASD

Based on the inclusion and exclusion criteria, a total of 129 patients with ASD (110 males and 19 females) were included in the analysis (Table 1). These 129 patients collectively completed 566 treatment courses. Specifically, 80 patients underwent four treatment courses, while 11 patients received seven courses. Additionally, 97 patients were treated with supplementary therapies such as behavioral, communication, or educational therapy, or medication, whereas 32 patients were exclusively administered WMT without additional interventions. Among the 566 WMT courses, only 14 resulted in adverse effects, which included fever (1.00%), diarrhea (0.50%), abdominal pain (0.70%), and vomiting (0.50%) (Table 2). All adverse effects were mild and resolved following symptomatic treatment, with no serious adverse events reported.

**Table 1:** Baseline characteristics of the patients with ASD.

Clinical characteristics	Patients with ASD (*n* = 129)
Age (years)	6.00 (4.00–8.00)
Male (%)	110 (85.20%)
BMI (kg/m^2^)	15.70 (14.54–17.73)
Disease duration (years)	3.25 (2.00–5.045)
ABC	67.00 (56.00–83.50)
CARS	36.00 (34.00–38.00)
SDSC	44.00 (36.00–53.00)
Constipation	48 (37.20%)
Diarrhea	6 (4.60%)
BSFS	40 (31.00%) (*n* = 128)
Treatment	
Behavioral intervention and drugs	9 (7.00%)
Behavioral intervention	88 (68.20%)
Drugs	1 (0.80%)
No treatment	31 (24.00%)

Data are presented as mean ± standard deviation, median (interquartile range), or *n* (%). ASD, autism spectrum disorder; BMI, body mass index; ABC, Aberrant Behavior Checklist; CARS, Childhood Autism Rating Scale; SDSC, Sleep Disturbance Scale for Children; BSFS, Bristol Stool Form Scale (abnormal stool form: scores of 1, 2, and 6; normal stool form: scores of 3, 4, and 5).

**Table 2:** Treatment-related adverse events observed during the washed microbiota transplantation procedure.

Adverse event	WMT course (*n* = 566)
Fever	6 (1.00%)
Diarrhea	3 (0.50%)
Abdominal pain	4 (0.70%)
Vomiting	3 (0.50%)
Overall	16%

A total of 566 microbiota transplantation procedures were conducted in 129 patients with ASD. Multiple adverse events were observed in some patients during a single treatment course. However, no patient experienced adverse events across two treatment courses.

### WMT significantly improved ASD symptoms

**Figure 1:**
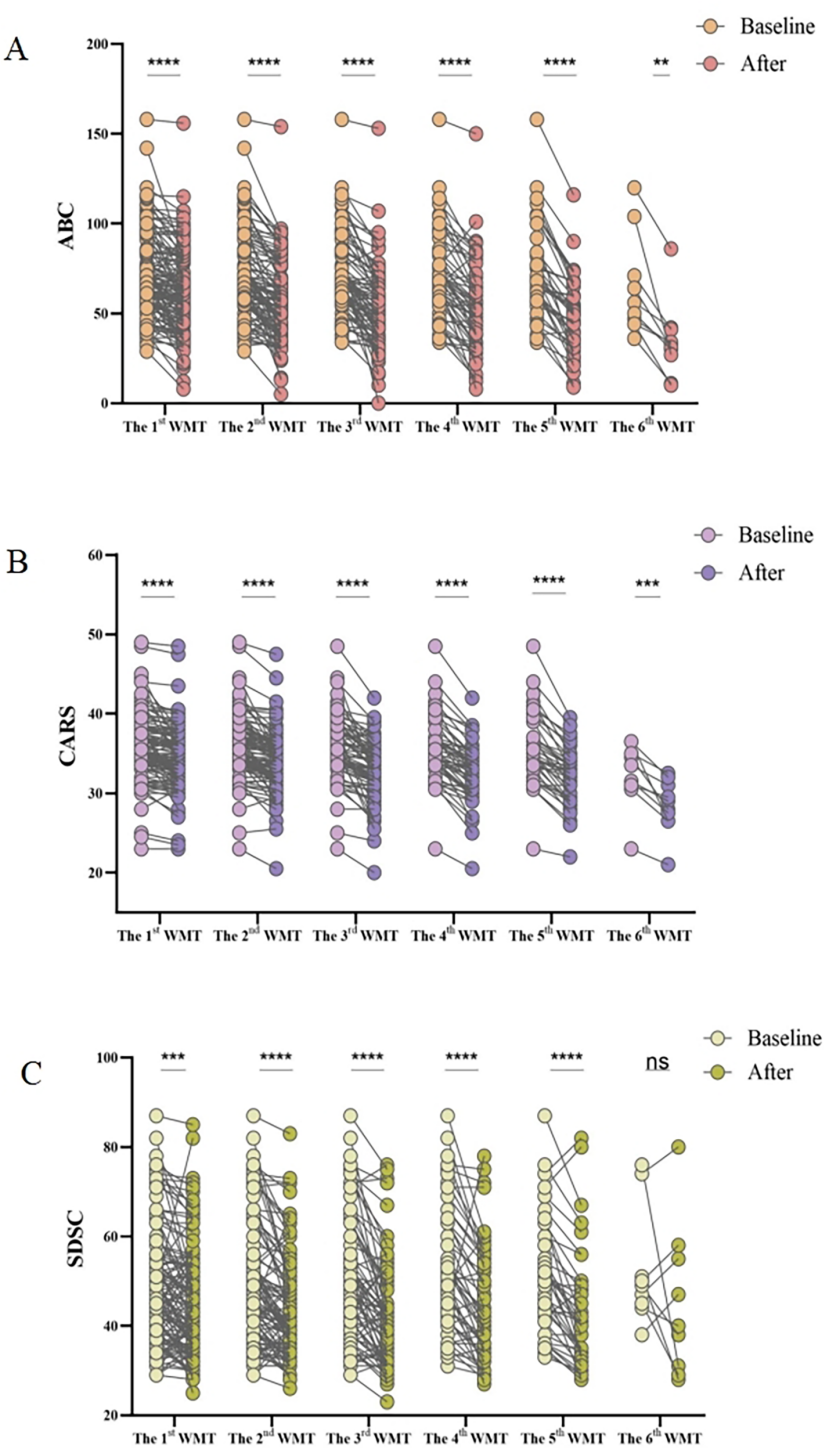
Effect of WMT on ASD symptoms and sleep disorders. Changes in the ABC **(A)**, CARS **(B)**, and SDSC **(C)** scores. Paired data obtained after each treatment course were compared with the primary baseline data. ABC, Aberrant Behavior Checklist; ASD, autism spectrum disorder; CARS, Childhood Autism Rating Scale; SDSC, Sleep Disturbance Scale for Children; WMT, washed microbiota transplantation. Statistical analysis was performed using the paired Student's *t*-test or the Wilcoxon signed-rank test. *Denotes statistical significance at *p* < 0.05; ** at *p* < 0.01; *** at *p* < 0.001; and **** at *p* < 0.0001. ^ns^indicates non-significance at *p* > 0.05.

The improvement in ASD symptoms after WMT was primarily assessed using the ABC, CARS, and SDSC. The post-treatment scores from these questionnaires were compared to baseline scores, as shown in Figure 1. The results demonstrated that WMT led to a significant reduction in the ABC (Figure 1A), CARS (Figure 1B), and SDSC scores (Figure 1C), indicating improvement in ASD symptoms.

**Figure 2:**
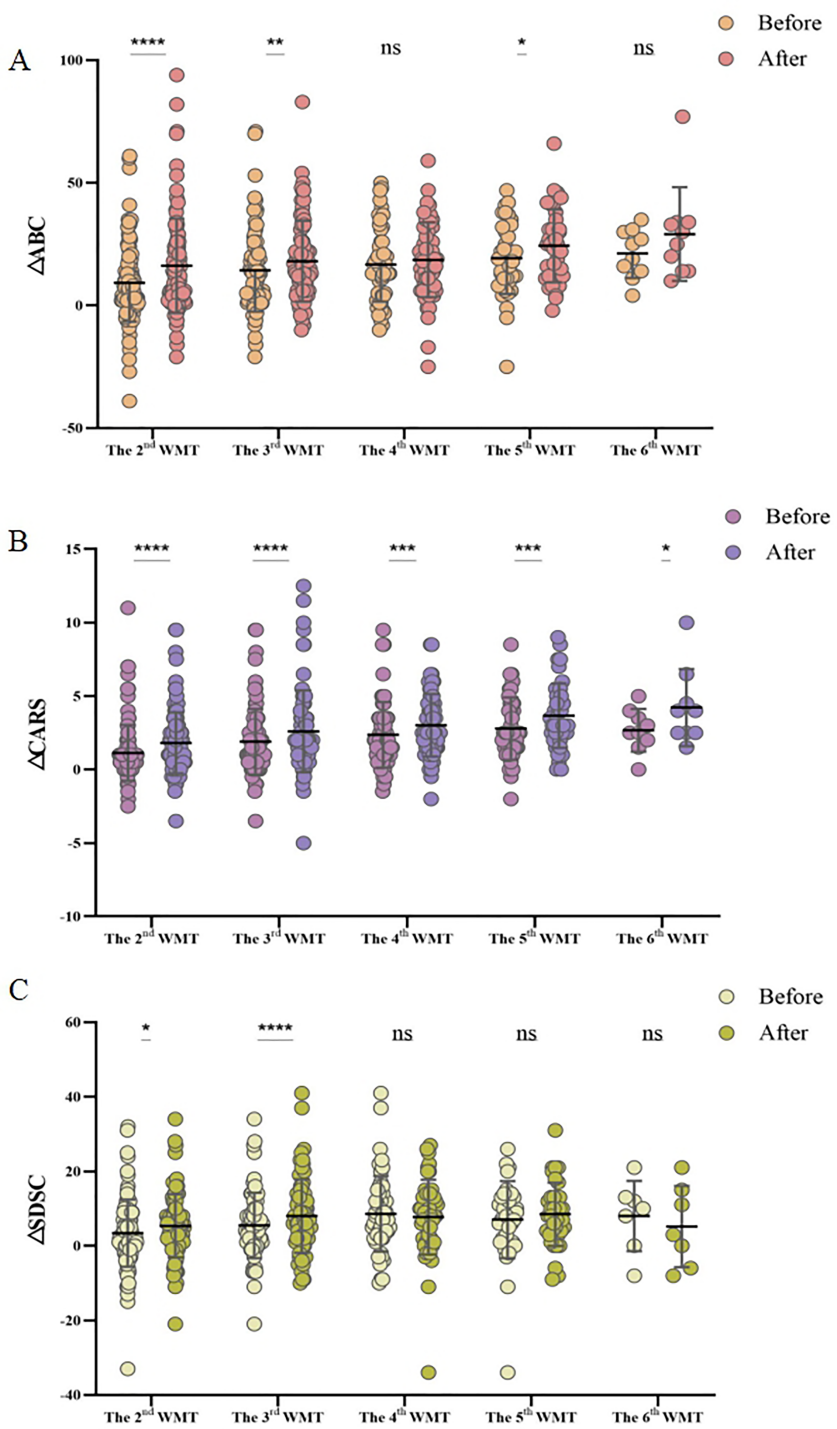
Effect of the number of WMT treatment courses on the ABC **(A)**, CARS **(B)**, and SDSC **(C)** scores. MABC: the difference between the ABC scores after WMT and at primary baseline; MCARS: the difference between the CARS scores following WMT and at primary baseline; and MSDSC: the difference between the SDSC scores after WMT and at primary baseline. ABC, Aberrant Behavior Checklist; CARS, Childhood Autism Rating Scale; SDSC, Sleep Disturbance Scale for Children; WMT, washed microbiota transplantation. The paired Student's *t*-test and Wilcoxon signed-rank test were employed for data comparison. Horizontal lines in the graphs represent the mean ± standard deviation. * denotes statistical significance at *p* < 0.05; ** at *p* < 0.01; *** at *p* < 0.001; and **** at *p* < 0.0001, ^ns^indicates non-significance at *p* > 0.05.

Furthermore, the relationship between the number of WMT courses and treatment efficacy was explored. Figure 2 illustrates the changes in questionnaire scores (ΔCARS, ΔABC, and ΔSDSC) before and after each increase in the number of WMT courses. The findings suggested that a greater number of treatment courses correlated with a more substantial reduction in symptom severity. Specifically, after six courses of WMT, there was a significant decrease in CARS scores, indicating that increasing the number of treatment courses led to more pronounced improvements in ASD symptoms (Figure 2A). For both the ABC and SDSC scores, significant reductions were observed after three courses, with further treatment leading to increasingly notable improvements (Figures 2B,C).

### WMT significantly altered GI symptoms

**Figure 3:**
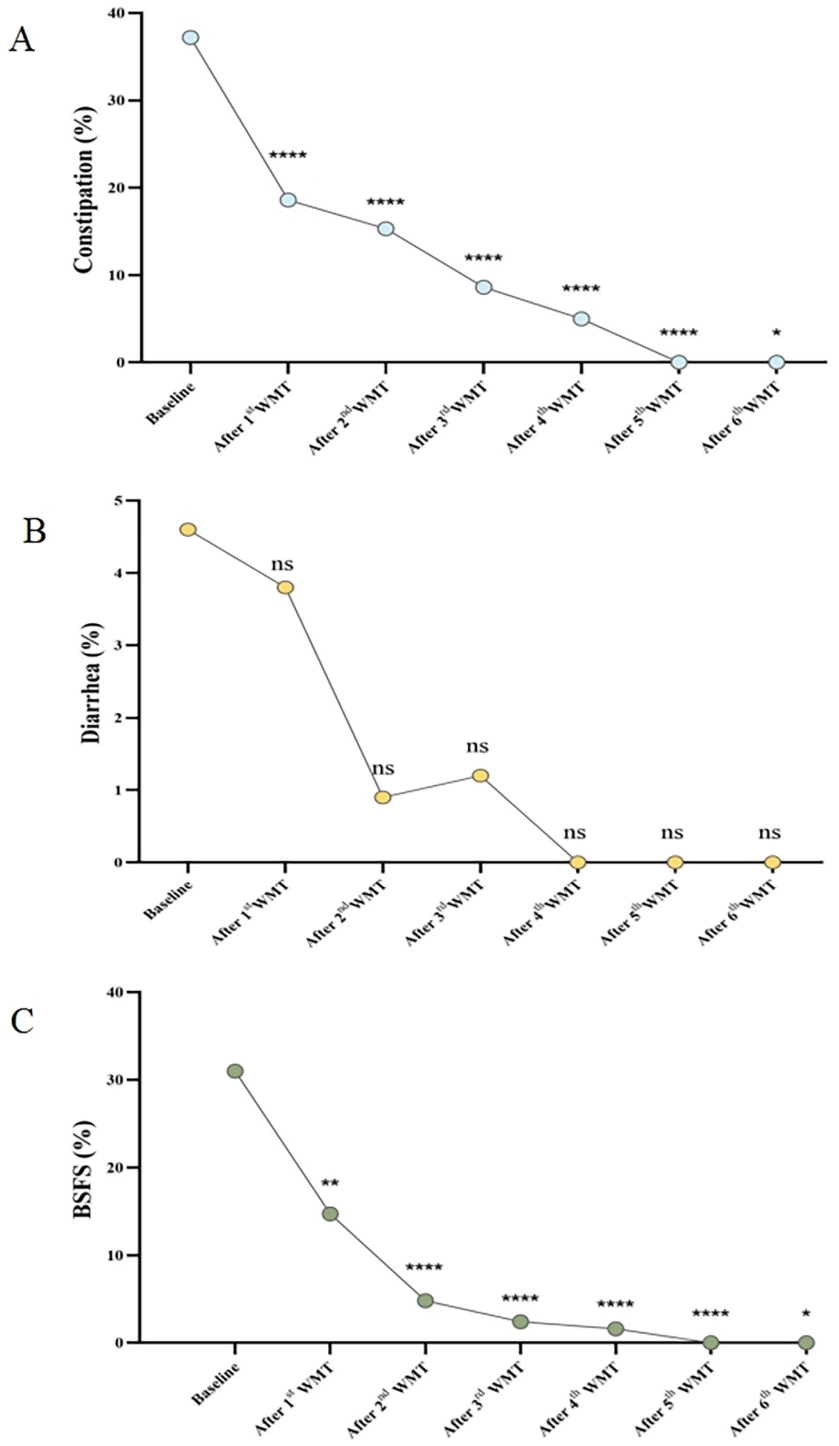
Effect of WMT on GI symptoms. **(A)** Changes in the proportion of patients with constipation. **(B)** Changes in the proportion of patients with diarrhea. **(C)** Changes in the proportion of patients with abnormal stool form according to the BSFS score. Data collected after each WMT treatment course were compared with the primary baseline data. WMT, washed microbiota transplantation; and BSFS, Bristol Stool Form Scale (abnormal stool form: scores of 1, 2, and 6; normal stool form: scores of 3, 4, and 5). Statistical significance was evaluated using the Wilcoxon signed-rank test, Fisher's exact test, and Chi-square test. *Denotes statistical significance at *p* < 0.05; ** at *p* < 0.01; *** at *p* < 0.001; and **** at *p* < 0.0001. ^ns^indicates non-significance at *p* > 0.05.

GI symptoms, such as constipation, diarrhea, and abnormal stool form, are common comorbidities in patients with ASD. To evaluate the effect of WMT on GI symptoms, the incidence of these symptoms before and after each treatment course was compared. Compared to the baseline incidence of constipation, the occurrence of constipation was significantly reduced after the first WMT course (0.37 vs. 0.17, *p* < 0.0001, Figure 3A). By the sixth treatment course, all patients were free of constipation. Similarly, the incidence of abnormal stool form significantly decreased after the first WMT course (0.31 vs. 0.15, *p* < 0.0001, Figure 3C). With increasing WMT courses, the proportion of abnormal stool form continued to decline. However, the proportion of patients experiencing diarrhea did not significantly change across the six treatment courses (Figure 3B).

### Effect of GI symptoms on the efficacy of WMT in the treatment of ASD

**Figure 4:**
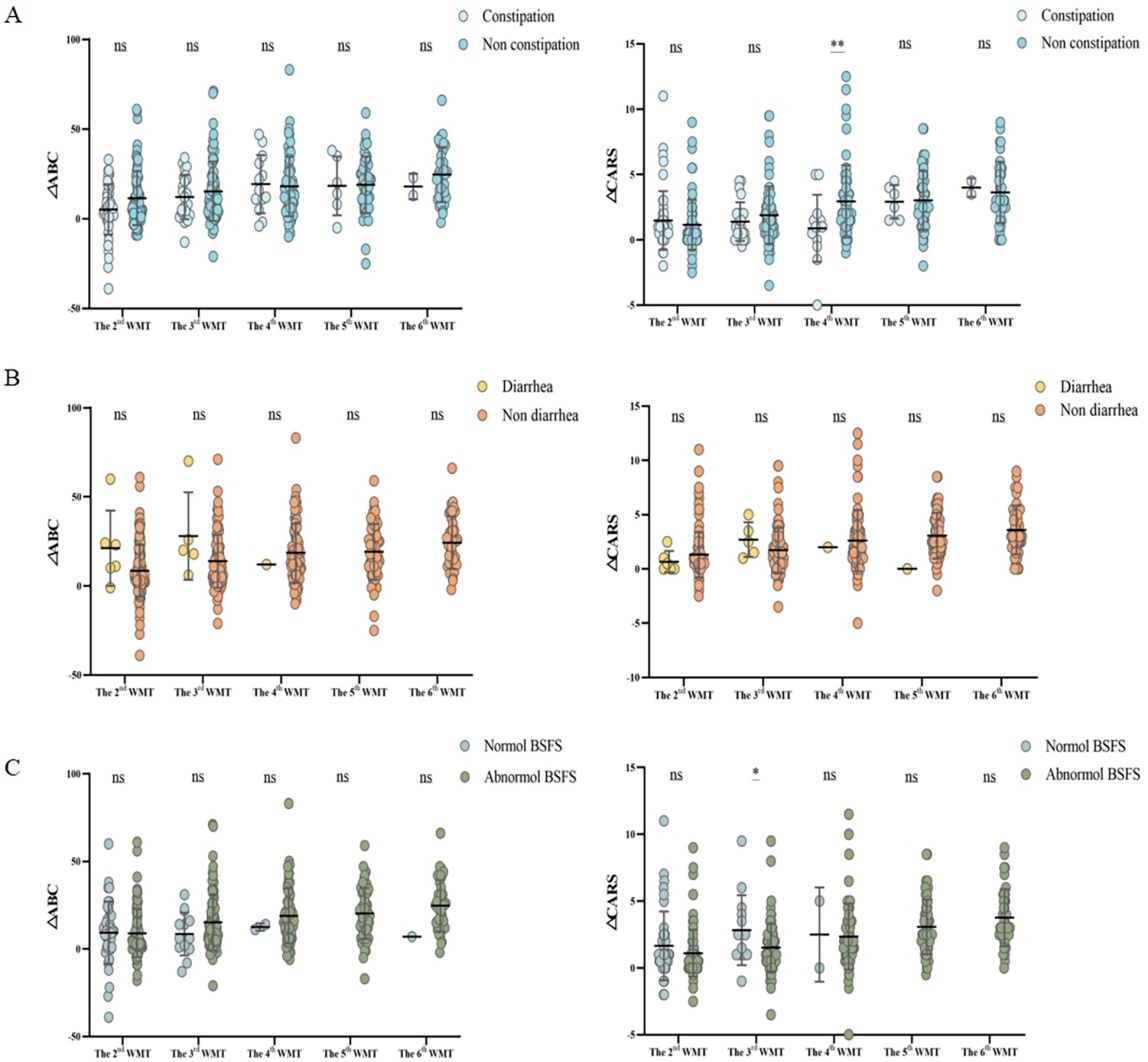
Effect of the number of WMT treatment courses on gastrointestinal symptoms according to the ABC and CARS scores. Changes in the proportion of patients with **(A)** constipation, **(B)** diarrhea, and **(C)** abnormal stool form (based on the BSFS score). ΔABC: the difference between the ABC scores after WMT and at primary baseline; ΔCARS: the difference between the CARS scores following WMT and at primary baseline; WMT, washed microbiota transplantation; ABC, Aberrant Behavior Checklist; CARS, Childhood Autism Rating Scale; and BSFS, Bristol Stool Form Scale (abnormal stool form: scores of 1, 2, and 6; normal stool form: scores of 3, 4, and 5). Student's *t*-tests were employed to compare the data. Horizontal lines in the graphs represent the mean ± standard deviation. * denotes statistical significance at *p* < 0.05; ** at *p* < 0.01; *** at *p* < 0.001; and **** at *p* < 0.0001. ^ns^indicates non-significance at *p* > 0.05.

To assess the impact of GI symptoms on WMT efficacy, changes in the ΔABC, ΔCARS, and ΔSDSC scores (Figure 4) were compared between patients with and without GI symptoms before and after each treatment course. The results indicated that patients without constipation showed a significantly better response to WMT. Although, in the univariate analysis, patients with diarrhea and abnormal stool form tended to exhibit greater improvement in autism symptoms compared to those without these symptoms, the differences were not statistically significant.

### Factors influencing the efficacy of WMT in the treatment of ASD

Multiple linear regression analyses were performed to evaluate the influence of various factors on WMT efficacy in ASD treatment (Tables 3, 4). The results revealed that patients with ASD and GI symptoms, such as diarrhea and abnormal stool form, demonstrated more pronounced therapeutic responses, as did those without constipation. Additionally, a positive correlation was found between the number of treatment courses and improved efficacy, with a greater number of WMT courses leading to better outcomes. Longer disease duration was also associated with enhanced efficacy. Moreover, patients who did not receive additional interventions and those with more severe symptoms tended to show better therapeutic responses. Males and patients with a lower BMI also exhibited relatively improved outcomes. However, age did not significantly influence WMT efficacy in ASD treatment. In summary, patients with ASD and co-existing GI symptoms prior to WMT treatment may experience more favorable outcomes.

**Table 3:** Multiple linear regression analysis of the influence of different factors on the efficacy of washed microbiota transplantation in patients with ASD based on their CARS scores.

Dependent variable	Independent variable	*β*	*p*
ΔCARS	Male	0.16	<0.001
	Age	−0.116	0.069
	BMI (kg/m^2^)	0.022	0.457
	Disease duration (years)	0.168	0.007
	Treatment course	0.204	<0.001
	Constipation	−0.025	0.429
	Diarrhea	0.03	0.308
	Abnormal stool form	0.364	<0.001
	Additional treatment	−0.08	0.009
	Disease severity	0.296	<0.001

ASD, autism spectrum disorder; WMT, washed microbiota transplantation; ΔCARS, the difference between the CARS scores after WMT and at primary baseline; BMI, body mass index; CARS, Childhood Autism Rating Scale; BSFS, Bristol Stool Form Scale (abnormal stool form: scores of 1, 2, and 6; normal stool form: scores of 3, 4, and 5). Severe symptoms are defined as a CARS score >36, while mild to moderate symptoms are indicated by a CARS score ≤36.

**Table 4:** Multiple linear regression analysis of the influence of different factors on the efficacy of washed microbiota transplantation in patients with ASD based on their ABC scores.

Dependent variable	Independent variable	β	*p*
ΔABC	Male	0.14	<0.001
	Age	0.047	0.082
	BMI (kg/m^2^)	−0.169	<0.001
	Disease duration (years)	0.1	<0.001
	Treatment course	0.116	<0.001
	Constipation	−0.179	<0.001
	Diarrhea	0.119	<0.001
	Abnormal stool form	0.201	<0.001
	Additional treatment	−0.041	0.002
	Disease severity	0.125	<0.001

ASD, autism spectrum disorder; WMT, washed microbiota transplantation; ΔABC, the difference between the ABC scores after WMT and at primary baseline; BMI, body mass index; ABC, Aberrant Behavior Checklist; BSFS, Bristol Stool Form Scale (abnormal stool form: scores of 1, 2, and 6; normal stool form: scores of 3, 4, and 5). Severe symptoms are defined as a CARS score >36, while mild to moderate symptoms are indicated by a CARS score ≤36.

## Discussion

This study demonstrates that WMT significantly improves both ASD-related symptoms and sleep disorders. Six courses of WMT were particularly effective in treating ASD, with the therapeutic effect improving as the number of courses increased. Notably, patients who did not receive additional interventions, as well as those with severe symptoms and prolonged disease duration, exhibited the most significant improvements. Additionally, WMT alleviated constipation, diarrhea, and abnormal stool form. To our knowledge, this study is the first to suggest that pre-treatment GI symptoms, including diarrhea and diarrhea abnormal stool form, may influence the efficacy of WMT in ASD therapy.

Recent research has recognized FMT as a promising therapeutic option for ASD treatment (32). An open-label study found that patients with ASD who received FMT exhibited significant improvements in their autism symptoms (33). Further studies have shown that microbiota transfer therapy can substantially reduce both GI and ASD symptoms, with these improvements persisting for at least 8 weeks post-treatment (9, 34). WMT, an enhanced version of FMT, has also been proven to significantly improve ASD, GI symptoms, and sleep disturbances (13, 14, 35). In line with these findings, our study adds to the growing body of evidence, offering validation with a larger sample size.

The duration of FMT has been previously linked to its efficacy. For instance, Hui et al. demonstrated that multiple FMT infusions significantly improved the clinical remission rate of diarrhea in patients with recurrent *Clostridium difficile* infection (CDI) (36). Similarly, multiple courses of WMT have been shown to outperform single or double courses in patients with hyperlipidemia (37). Additionally, a study indicated that three FMT courses led to significant improvements in ASD-related symptoms and sleep disorders, with additional courses further improving outcomes (13). Consistent with these studies, our research found that WMT was effective after six courses, with treatment benefits increasing as the number of courses grew. These results underscore the potential for improved outcomes in patients adhering to FMT-based therapies. However, further studies are needed to determine the optimal number of treatment courses for maximal benefit.

Gut microbiota may influence autism symptoms through several mechanisms. Notably, gut microbiota modulates social behavior in individuals with autism via neurotransmitters, particularly 5-hydroxytryptamine (5-HT), a monoamine neurotransmitter that regulates mood, behavior, and neural development (38, 39). Xiao et al. observed that 5-HT levels were elevated in fecal samples from GF mice following FMT (40). Proinflammatory cytokines such as interleukin-6 (IL-6) and tumor necrosis factor-α (TNF-α) have been implicated in the development of severe ASD-related neurobehavioral symptoms (41, 42), with elevated peripheral blood levels observed in patients with ASD (43). Avolio et al. demonstrated that FMT reduced IL-6 and TNF-α levels in the gut and brain of autism mouse models (44). Additionally, fecal samples from individuals with autism exhibit reduced levels of short-chain fatty acids (SCFAs) (45), influencing brain development and social behavior (46). Several studies have shown that FMT significantly increases SCFA levels in patients with ASD (47).

GI symptoms, including constipation, diarrhea, and abnormal stool form, are prevalent in individuals with ASD (7, 48). Numerous clinical studies have shown that FMT not only alleviates autism symptoms but also significantly improves GI symptoms (9, 33, 49). Consistent with these findings, the present study demonstrates that WMT improves GI symptoms in patients with autism. However, the precise mechanisms by which WMT ameliorates GI symptoms remain unclear. Both inflammatory responses and intestinal barrier dysfunction are believed to contribute to these symptoms. FMT has been shown to reduce serum IL-6 levels in patients with UC, suggesting its potential to mitigate inflammatory diseases by decreasing cytokine-mediated inflammation (50). Furthermore, FMT reduces intestinal inflammation and enhances intestinal barrier function, which helps alleviate CDI-induced diarrhea (51). These mechanisms may similarly underlie the therapeutic effects of WMT in improving GI symptoms in patients with autism.

Although FMT has been established as an effective treatment for ASD, its therapeutic efficacy can vary, resulting in differing clinical outcomes among patients (9, 52). Previous short-term WMT studies reported better outcomes in patients without constipation (35), aligning with our findings that increased treatment courses lead to improved efficacy. Many studies have also indicated that the effectiveness of FMT is influenced by the disease duration and treatment period. For instance, Moayyedi et al. demonstrated that FMT was more beneficial in patients with a shorter duration of UC (23). In contrast, a longer disease duration in patients with autism corresponded to improved efficacy. Given the potential for delayed autism diagnosis, this observation warrants further exploration. Furthermore, multiple courses of WMT have been found to be more effective than single or double courses in treating patients with hyperlipidemia (37). Numerous studies have highlighted a strong correlation between GI symptoms and ASD severity (53, 54), with other research showing that patients with GI symptoms tend to have higher CARS and ABC scores (33), reflecting more severe disease. Additionally, WMT was shown to significantly reduce the incidence of diarrhea in patients with CDI (55) and markedly improve abnormal stool form in patients with ASD (33). These findings suggest that WMT may alleviate autism symptoms by improving GI function.

However, several limitations remain. First, the potential influence of confounding variables, such as genetic factors related to autism and the varying cognitive functioning of patients (high-functioning vs. low-functioning), were not considered, which may have affected WMT's efficacy in treating autism. Secondly, while animal and clinical studies have demonstrated that FMT enhances short-chain fatty acid production and reduces inflammatory markers, the impact of FMT on other metabolites or proteomic elements remains uncertain, due to the insufficient fecal samples for analysis in our study. Lastly, the study was limited by its small sample size and single-center design. Therefore, larger, multicenter studies are needed to validate our findings.

## Conclusion

In conclusion, this study demonstrates that WMT can significantly improve ASD, GI symptoms, and sleep disturbances in patients with ASD. Six courses of WMT show enhanced therapeutic outcomes, with efficacy improving as the number of courses increases. Patients without additional treatments, as well as those with more severe conditions and longer disease durations, exhibited better outcomes. Additionally, pre-treatment GI symptoms, such as abnormal stool form and diarrhea, may influence the efficacy of WMT in patients with ASD.

## Data Availability

The raw data supporting the conclusions of this article will be made available by the authors, without undue reservation.
